# Integrated Limb Lengthening Is Superior to Classical Limb Lengthening: A Systematic Review and Meta-analysis of the Literature

**DOI:** 10.5435/JAAOSGlobal-D-20-00054

**Published:** 2020-06-12

**Authors:** Gerard A. Sheridan, Austin T. Fragomen, S. Robert Rozbruch

**Affiliations:** From the Limb Lengthening and Complex Reconstruction Service, Hospital for Special Surgery, New York, NY.

## Abstract

Integrated limb lengthening combines both internal and external fixation methods. It has been introduced to improve time to union, patient time in frame, risk of regenerate refracture, and patient function. We systematically review studies to assess whether integrated limb lengthening methods are superior to classic limb lengthening.

**Methods:**

A total of 457 patients had classic limb lengthening, whereas 488 underwent integrated limb lengthening. The primary outcome measures were total length achieved (cm), external fixator index (month/cm) and bone healing index (month/cm). Problems, obstacles, and sequelae were compared using random effects meta-analyses of all available cases. Kaplan-Meier curves were generated to compare the time spent in frame.

**Results:**

Integrated limb lengthening demonstrated a superior external fixator index (*P* = 0.0001) and bone healing index (*P* = 0.0146). The mean time spent in frame for integrated lengthening was significantly shorter (*P* = 0.0015). Significantly fewer problems (*P* = 0.000) and sequelae (*P* = 0.001) were observed with integrated lengthening. Deep infections were more common in the integrated cohort. The lengthening over a nail deep infection rate was significantly higher than with the lengthening and then nailing and lengthening and then plating techniques (*P* = 0.005).

**Conclusions:**

Integrated methods of limb lengthening are superior to classic methods. We suggest the integration of plates and nails with circular frames to improve outcomes in patients undergoing limb lengthening procedures.

Distraction osteogenesis (DO) has been successfully used over the past half century to lengthen bone. In 1905, Professor Codivilla^1^ began an investigation into the lengthening of bone to treat deformity and malunion. Since then, the field has advanced in myriad ways, although the most effective techniques regarding the process of DO were not always well known.^2^ Critical to successful DO is an optimal rate and rhythm of distraction and ideal stability for which external fixation has been a reliable tool. External fixation alone has become the benchmark for providing stability with this technique, a technique known as the “classical method” or “Ilizarov method.” Ilizarov pioneered many advancements in the field, after he began his experimentation with external fixators in the 1950s.^3–5^ Others have also refined the desirable DO characteristics to include younger patients, a metaphyseal osteotomy and a double-level corticotomy.^6^

The Ilizarov method has demonstrated excellent outcomes, but the reliance on external fixation has come into question in recent times, initiating a search for techniques that may minimize the need for external fixation when performing DO. The disadvantages of external fixators are well known to include pin tract infections, skin pain, soft-tissue tethering, and joint stiffness. Integrated fixation techniques combining internal and external fixation such as lengthening over a nail (LON), lengthening and then nailing (LATN), lengthening and then plating (LATP), and bone transport over a nail were implemented to minimize the time in external fixation.

Building on these incremental advances, bone lengthening with a fully implantable device most recently has enabled us to entirely avoid external fixation in many cases.^7^ Still many situations exist where internal lengthening nails are not indicated however, and so the decision of when to use integrated or classic techniques still remains relevant. In the following study, we explore the advantages of integrated limb lengthening over classic limb lengthening regarding a number of pertinent outcome measures.

## Methods

### Eligibility Criteria

**Figure 1:**
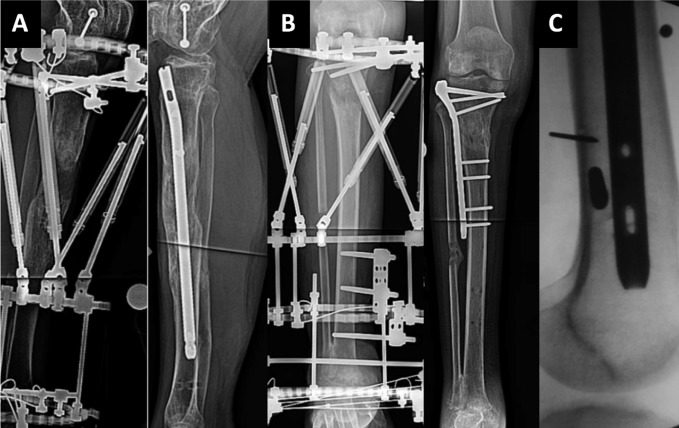
Radiographs demonstrating (**A**) lengthening and then nailing and (**B**) lengthening and then plating techniques. **C,** Fluoroscopic image demonstrating lengthening over nail with external pins avoiding the intramedullary nail.

Only cohort studies directly comparing classic limb lengthening and integrated limb lengthening techniques were included in the systematic review. “Integrated” limb lengthening was deemed to include the following techniques: LATN, LON, and LATP (Figure 1). A minimum of 9 months follow-up was required for inclusion. A minimum data set included external fixation index (EFI) (month/cm), bone healing index (BHI) (month/cm), total lengthening (cm), total time in frame (weeks), and all-cause revision details. Details pertaining to problems, obstacles, and sequelae were preferred but were not an absolute indication for inclusion. Only articles in the English language were considered. The PRISMA guidelines were adhered to throughout this study.^8^

### Search Strategy

**Figure 2:**
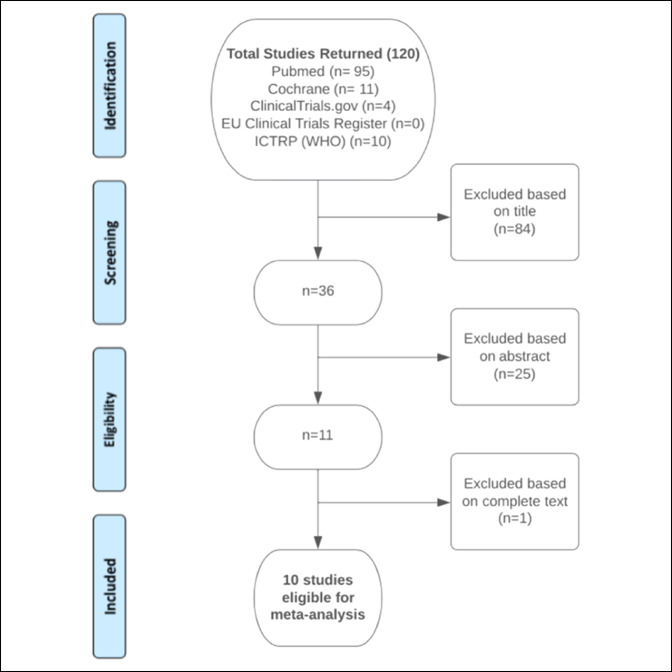
Flow diagram representing PRISMA. ICTRP = International Clinical Trials Registry Platform, WHO = World Health Organization

On December 9, 2019, a number of electronic bibliographic databases and clinical trial registries were searched using the following MeSH terms: “limb lengthening,” “Ilizarov,” “lengthening and then nail,” “LATN,” “lengthening over nail,” “LON,” “lengthening and then plate,” “LATP,” “external fixator index,” and “bone healing index” in various combinations to return a maximal number of studies for review. Locations searched included PubMed, the Cochrane Library, ClinicalTrials.gov, the European Union clinical trials register, and the International Clinical Trials Registry Platform (World Health Organization). The results were assessed on two separate occasions to ensure accuracy of the returned results. Studies were examined for eligibility based on the title initially. Abstracts were then reviewed, and studies were excluded based on the abovementioned inclusion criteria (Figure 2). Final review of full articles was performed by the authors, and any contention was resolved through consensus with all authors. Study selection was unblinded.

### Data Extraction

Relevant data were extracted using an electronic data extraction form. Extracted information included the following: author, year, journal, country of origin, total limbs, total patients, number of classic lengthenings, number of integrated lengthenings (LATN, LON, and LATP), mean follow-up, sex, age, indication for lengthening, implant specifications, EFI, BHI, time in frame, complications (problems, obstacles, and sequelae), indication for revision, and time to revision (Table 1).

**Table 1:** Minimum Data Set

Study	Journal	Total Classic Limbs	Total Integrated Limbs	Integration Technique	Minimum Follow-up (months)	Mean Age (Classic: Integrated)	Length Achieved (Classic: Integrated)	Classic EFI (months/cm)	Integrated EFI (months/cm)	Classic BHI (months/cm)	Integrated BHI (months/cm)
Rozbruch et al^12^	*Clinical Orthopaedics and Related Research*	34	39	LATN	11.4	30: 35	3.9 cm: 5.4 cm	1.9	0.5	1.9	0.8
Lan et al^13^	*International Orthopaedics*	98	78	LATN	12	25.7: 26.8	9.3 cm: 8.5 cm	2.5	0.7	2.53	1.44
Paley et al^14^	*The Journal of Bone and Joint Surgery (Am)*	32	32	LON	24	25: 26	5.2 cm: 5.8 cm	1.7	0.7	1.7	1.4
Park et al^15^	*The Journal of Bone and Joint Surgery (Am)*	32	56	LON	29	23.4: 22.3	5.9 cm: 6.4 cm	2.2	0.9	2.1	1.7
Sun et al^16^	*The Journal of Bone and Joint Surgery (Br)*	146	143	LON	14.9	21.2: 23	7 cm: 7.95 cm	1.3	1.1	1.8	1.5
Guo et al^17^	*International Orthopaedics*	23	51	LON	22	22.7: 25.4	7.2 cm: 7.4 cm	1.3	0.58	1.35	1.36
El-Husseini et al^18^	*Strategies in Trauma and Limb Reconstruction*	16	15	LON	12	28.4: 31.3	4.98 cm: 4 cm	1.2	0.4	1.2	1.4
Burghardt et al^19^	*Bone and Joint Research*	19	19	LON	9	27: 27	4.9 cm: 5.2 cm	2.3	0.7	2.3	1.4
Harbacheuski et al^20^	*Clinical Orthopaedics and Related Research*	27	27	LATP	25	41.1: 41.3	3.5 cm: 3.6 cm	2	1.3	2.2	2.1
Bernstein et al^21^	*Clinical Orthopaedics and Related Research*	30	28	1 LON 16 LATN 11 LATP	12	43: 48	5.3 cm: 4.4 cm	2.5	2	—	—

BHI = bone healing index, EFI = external fixation index, LATN = lengthening and then nailing, LATP = lengthening and then plating, LON = lengthening over nail

### Statistical Analysis

Statistical software (Stata/IC 13.1 for Mac [64-bit Intel]) was used to conduct all statistical analyses. Initial demographic data were detailed with descriptive statistics. The primary outcome measures were total length achieved (cm), time in frame (weeks), all-cause revision, EFI (month/cm), and BHI (month/cm). Kaplan-Meier curves were generated to compare the time spent in frame for both methods of limb lengthening. The two-sample Student *t*-test with equal variances was used to detect a notable difference between both groups. Boxplot graphs were used to demonstrate the interquartile distributions of EFI and BHI for both classic and integrated limb lengthening techniques using a two-sample Student *t*-test with equal variances.

Secondary outcomes including problems, obstacles, and sequelae as described by Paley et al^9^ were compared using a random effects meta-analysis of all available cases. The “metan” command was used to perform the random effects meta-analysis.^10^ The relative risk (RR) for the relevant outcome measure was calculated with 95% confidence intervals (CIs), and a percentage weight was attributed. The results are illustrated on a forest plot graph. The horizontal line width for each study represents the 95% CI, with the central square area being proportional to the weight of each individual study. Studies with a line width traversing the value one were deemed inconclusive. The accumulated 95% CI for all studies is represented by the width of the diamond which represents all studies overall. A *P* value of less than 0.05 was taken to be statistically significant when analyzing RRs. The contribution of potential inter-study heterogeneity was analyzed using the chi-squared test and the *I*^2^ statistic. Variation in RR because of heterogeneity was expressed as a percentage, and a *P* value >0.05 inferred that heterogeneity had no significant impact on the results described. The presence of deep infection for each study was recorded as a binary outcome (present or absent). The integration method used was then analyzed as a predictor for infection using technique as an independent variable and deep infection as the dependent variable. The Fisher exact test was used to determine significant correlation between fixation techniques and deep infection rates in this regard, again with a *P* value <0.05 taken to be clinically significant.

### Bias

**Figure 3:**
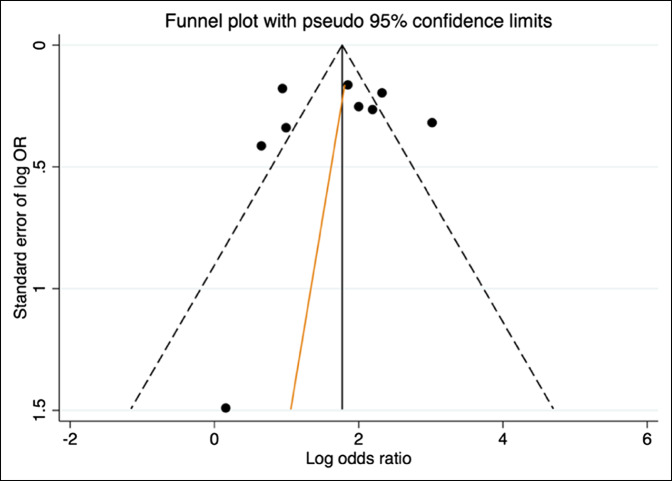
Funnel plot diagram illustrating no small study effects.

To eliminate the effect of publication bias, the effects of small studies were analyzed visually through the use of a funnel plot (Figure 3). To assess the funnel plot for statistically notable asymmetry, the Egger test for small-study effects was used.^11^ Again, a *P* value of <0.05 was taken to be significant.

### Study Results

As detailed in Figure 2, 120 studies were originally identified after searching all relevant databases with our specified MeSH terms. Eighty-four studies were deemed inappropriate for inclusion based on the title. A further 25 were excluded based on the abstract. A further study was excluded based on review of the full text of the study. In total, 110 of the original 120 studies failed to meet our inclusion criteria, leaving 10 well-designed comparative studies for systematic review and meta-analysis.

We found no evidence of publication bias on review of our funnel plot (Figure 3). The Egger test for small-study effects showed no statistically significant asymmetry in the funnel plot (*P* = 0.829). We can conclude, therefore, that no evidence of publication bias exists impacting on the validity of our findings.

## Results

Ten studies originating from four countries were included (USA,^5^ China,^2^ South Korea,^2^ and Egypt^1^). A total number of 457 limbs were lengthened using the classic technique, and 488 were lengthened using integrated techniques. The commonest method of integrated lengthening was LON (n = 317), followed by LATN (n = 133) and LATP (n = 38). Minimum follow-up ranged from 9 to 29 months. The mean patient age in eight studies was in the third and early fourth decade of life.^12,13,14,15,16,17,18,19^ Only two studies reported mean ages in the fifth decade.^20,21^ In the classic group, a 60% male majority was noted. In the integrated group, a 54.3% male majority was noted.

The indications for limb lengthening were very diverse for both groups. All indications for lengthening could be organized under one of the following headings: congenital, developmental, post-traumatic, and constitutional short stature. The two studies by Lan et al^13^ and Park et al^15^ were the only studies where limb lengthening was indicated exclusively for constitutional short stature. Bernstein et al ^21^ did limb lengthening exclusively for post-traumatic bone loss. The seven other studies all had combinations of congenital, developmental, and post-traumatic indications for limb lengthening. Regarding implant specifications, the Taylor Spatial Frame (Smith & Nephew) was the external fixator of choice in three studies.^12,20,21^ Sun et al opted for hybrid external fixation, whereas the remaining six studies used an Ilizarov external fixator. For the integrated LON and LATN cohorts, a variety of different intramedullary (IM) nails were used. In both studies comparing LATP with classic lengthening, a locking plate (Smith & Nephew) was used for stabilizing the regenerate postlengthening.

### Total Length Achieved

The total length achieved in the integrated cohort exceeded that of the classic cohort in all studies except for three (Table 1).^13,18,21^ Lan et al ^13^ reported a length increase of 9.3 cm in the classic group compared with 8.5 cm in the integrated group. El-Husseini et al^18^ reported a 4.98 cm increase in length compared with a 4 cm increase in the integrated cohort. Bernstein et al reported a 5.3 cm increase in the classic group compared with a 4.4 cm increase in the integrated group. Weighted mean values gave an overall increase of 0.85 cm in total lengthening for the classic group compared with the integrated group for these 3 studies.

Total lengthening in the integrated cohort ranged from 0.1 to 1.5 cm higher than the classic cohort for the remaining 7 studies. Weighted mean values gave an overall increase of 0.55 cm in total lengthening for the integrated group compared with the classic group for these 7 studies.

### Time in Frame

**Figure 4:**
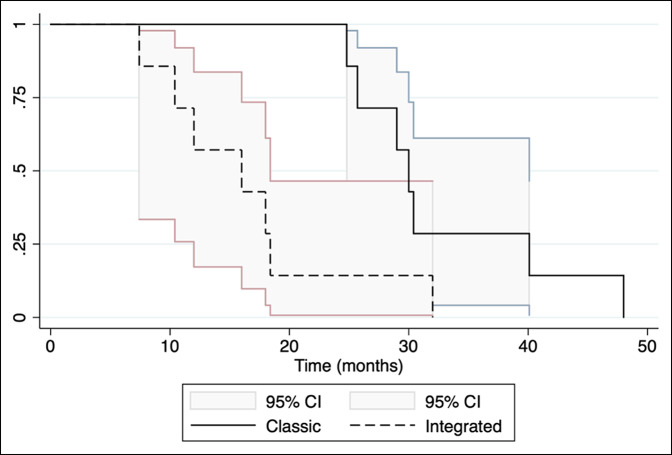
Time in frame (weeks) (Kaplan-Meier curve). CI = confidence interval

Using Kaplan-Meier survival estimates, with frame removal as the end point, we noted that the mean time in frame for the classic group was twice as long as the integrated group. The mean time spent in frame for the classic group was 32.6 weeks (σ = 8.43, 95% CI, 24.7 to 40.3), whereas the integrated group spent a mean time of 16.3 weeks (σ = 8.02, 95% CI, 8.9 to 23.7) in frame. A statistically significant difference was observed between the two groups in this regard (*P* = 0.0015) (Figure 4).

### All-cause Revision

In the classic group, 92 patients underwent revision surgery for numerous indications. The replacement of broken wires was not considered under the remit of revision surgery. Regarding delayed consolidation in the classic group, Lan et al ^13^ reported 6 cases needing revision surgery with iliac crest autograft. Paley et al ^14^ reported five delayed consolidations requiring revision. Park et al^15^ reported five and Sun et al^16^ reported four delayed consolidations, all requiring revision surgery. Ten premature consolidations requiring revision surgery were noted in three studies.^13,14,16^ Eleven cases of regenerate axial deviation were observed, requiring revision.^14,15,20^

The integrated group had a number of revisions for numerous indications, most notably: 10 premature consolidations^14,15^ and 12 delayed consolidations.^16^ Harbacheuski et al reported two regenerate collapses that were managed with a plate unbending in one case and Taylor Spatial Frame application in another case. These were the only two recorded cases of regenerate deviation in the integrated group. Unlike the classic group, a number of integrated patients had revision surgery for problematic internal hardware: 10 cases of painful hardware,^12^ a broken nail and prominent locking screw,^14^ 1 broken IM nail,^17^ 1 IM nail deep infection,^18^ 1 broken distal screw and 1 loose screw,^19^ and 2 plate fractures. In total, 18 patients underwent revision surgery for problematic internal hardware in the integrated group.

### External Fixator Index

**Figure 5:**
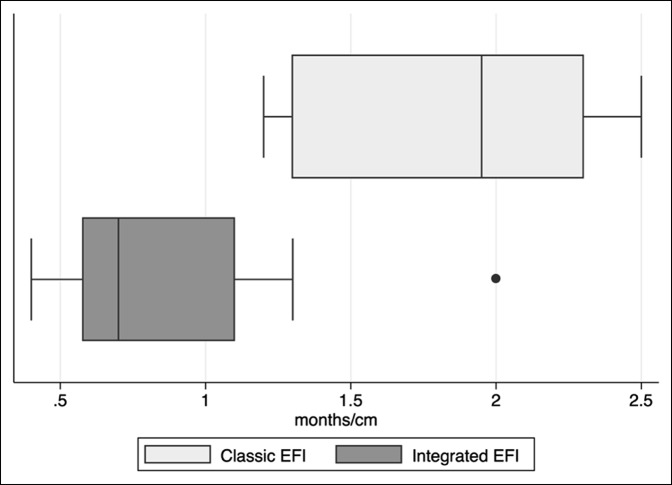
Box plots illustrating classic and integrated external fixation index (EFI) values.

The EFI for the integrated cohort was 0.88 months/cm (σ = 0.476, 95% CI, 0.547 to 1.22) compared with 1.89 months/cm in the classic cohort (σ = 0.497, 95% CI, 1.53 to 2.25). This difference was statistically significant (*P* = 0.0001) and is illustrated in Figure 5.

### Bone Healing Index

**Figure 6:**
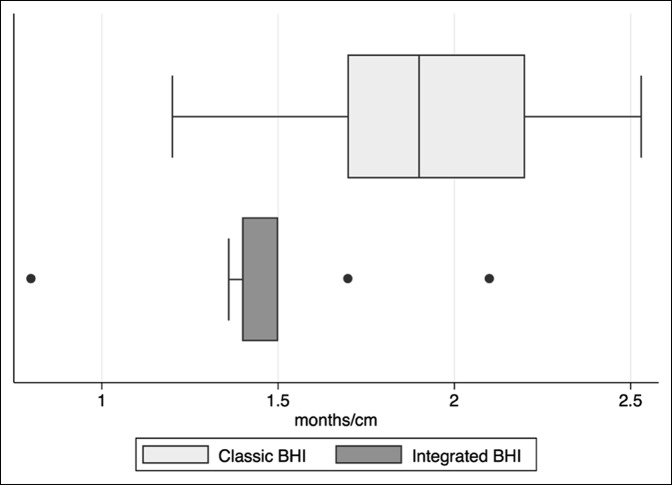
Box plots illustrating classic and integrated bone healing index (BHI) values.

The BHI for the integrated cohort was 1.45 months/cm (σ = 0.340, 95% CI, 1.194 to 1.716) compared with 1.90 months/cm in the classic cohort (σ = 0.437, 95% CI, 1.56 to 2.23). This difference was statistically significant (*P* = 0.0146) and is illustrated in Figure 6.

### Complications (Problems, Obstacles, and Sequelae)

The complication of deep infection is most interesting in this study because the common conception of increased deep infection rates with integrated lengthening has long been suspected. We detail in Figure 1, the importance of ensuring that both internal and external hardware structures are discreet from each other and do not contact directly to minimize inoculation of deep internal hardware that may in turn lead to osteomyelitis. In the classic group, we report four cases of deep bone infection and one septic arthritis of the knee.^12,13,20,21^ In the integrated group, we report nine cases of deep bone infection with one septic arthritis of the knee.^14,18,19,20,21^ Of note, all deep infections in the integrated group were recorded in the LON group with no deep infections recorded in the LATN or LATP groups. The presence of deep infection in the LON group was significantly higher than the LATN and LATP groups (*P* = 0.005).

To simplify the analysis of all complications, the classification described by Paley et al ^14^ detailing all complications as either problems, obstacles, or sequelae was adopted. Studies were excluded from the meta-analysis if they did not report on either the problems, obstacles, or sequelae.

**Figure 7:**
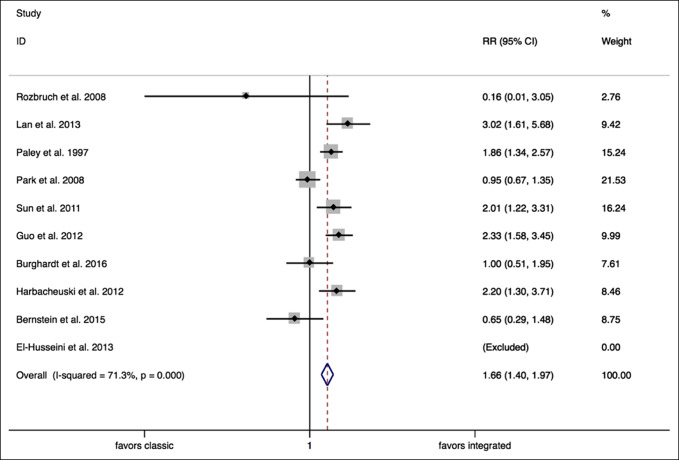
Forest plot (problems). CI = confidence interval, RR = relative risk

A random effects meta-analysis analyzing “problems” encountered confirmed a significantly higher RR of problems with classic lengthening techniques when compared with integrated lengthening techniques (RR = 1.66, 95% CI, 1.40 to 1.97, *P* = 0.000) (Figure 7).

**Figure 8:**
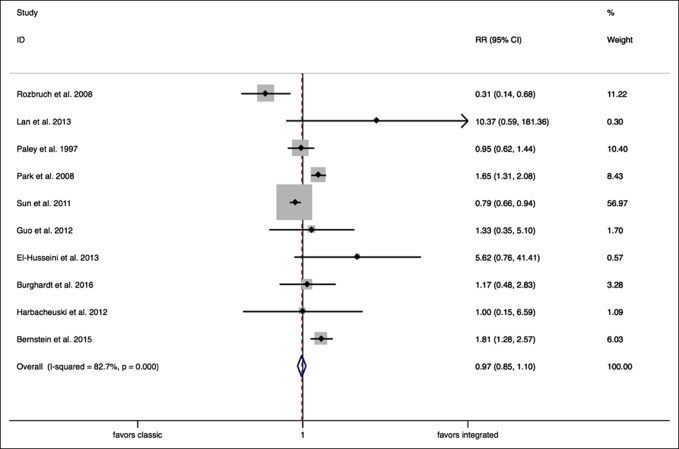
Forest plot (obstacles). CI = confidence interval, RR = relative risk

Random effects meta-analysis analyzing “obstacles” encountered showed no difference between classic lengthening techniques when compared with integrated lengthening techniques (RR = 0.97, 95% CI, 0.85 to 1.10, *P* = 0.621) (Figure 8).

**Figure 9:**
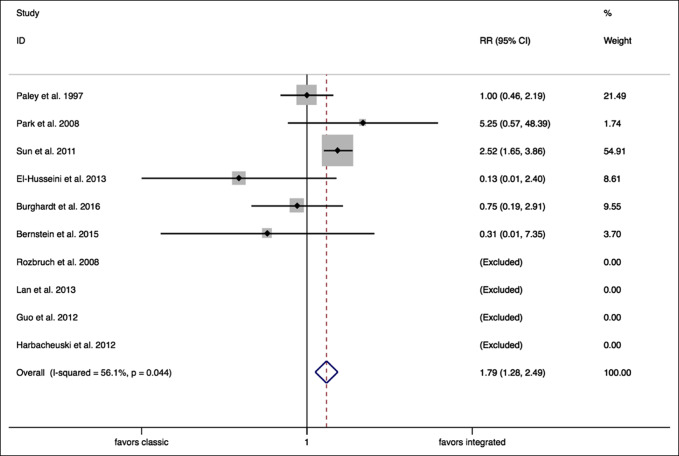
Forest plot (sequelae). CI = confidence interval, RR = relative risk

A random effects meta-analysis analyzing “sequelae” encountered confirmed a significantly higher RR of sequelae with classic lengthening techniques when compared with integrated lengthening techniques (RR = 1.79, 95% CI, 1.28 to 2.49, *P* = 0.001) (Figure 9).

## Discussion

Our results expand on the concept that an increasing construct stability can improve the efficacy of DO in limb lengthening. We see that the use of additional prostheses such as intramedullary nails, inserted during or after the process of lengthening, can notably reduce the EFI, the BHI, and the time a patient spends in frame. We also found that the specific type of integrated device used was not as important as the fact that an integrated technique of any kind was used. In our subgroup analysis, we observed that LON, LATN, and LATP techniques all demonstrated improved EFI, BHI, and time spent in frame in their own right. In a sample of 54 limbs, the lowest EFI was noted in the LATP group with a mean value of 1.3 months/cm.^20^ With a BHI of 0.8 months/cm, LATN was found to be most effective in this respect. The least time spent in frame was seen in the LATN group with a mean time of just 12 weeks. This demonstrates a 17-week reduction compared with their classic lengthening counterparts in this study. The LON technique was close behind with a mean time in frame of 13 weeks.

Overall, with all techniques included, the EFI was reduced by 1.01 months/cm and the BHI was reduced by 0.45 months/cm (*P* = 0.0001, *P* = 0.0146, respectively). With evolving surgical techniques and rising patient expectations, increased DO efficiency and reduction of the required time that a patient must spend in an external fixator is highly desirable. We demonstrate that integrated methods of lengthening halve the time a patient spent in an external fixator from 32.6 to 16.3 weeks (*P* = 0.0015). This is the first time that this critical improvement in the patient experience has been illustrated through the use of systematic review and meta-analysis.^22^

The reason behind the notably improved outcomes observed with integrated lengthening may stem from a number of factors known to be important in the DO process. Consider first, the role of angiogenesis in the process of active distraction. During DO, increased levels of angiogenic factors including angiopoietin 1 and 2, Tie receptors, VEGF-A and -D, VEGFR2, and neuropilin 1 have all been demonstrated through maximal mRNA expression.^23^ Angiogenesis is essential for new bone formation, and so any technique that improves angiogenesis is likely to improve bone consolidation that will then translate in to lower EFI, BHI, and time spent in frame. We know that reaming is an important step in the integrated lengthening philosophy and has been associated with increased periosteal bone formation in rabbit models and increased numbers of bone nodules in murine models.^24,25^ Reaming has also been shown to induce the level of IL-10 which is known to be proangiogenic.^26,27^ The technique of reaming may therefore accelerate the rate of bone consolidation through its effect on angiogenesis. This may prove to be essential in explaining the notable clinical improvements seen with integrated lengthening in this study.

Second, considering that some nails were introduced without reaming, we should explore other explanations for the success of integrated techniques. Both unreamed nails and LATP techniques do not induce angiogenesis and bone formation through reaming via the above described pathways. Both, however, do produce similar improvements in outcomes recorded in this study. It is reasonable to assume that the additional mechanical stability afforded by an unreamed nail or plate inserted after lengthening could explain the notable improvement in the EFI, BHI, and time in frame for the integrated cohort. We know from the original work of Ilizarov 4 that increased fixator stability enhanced bone formation during limb lengthening. Subsequent human clinical trials confirmed these findings and so strengthen the overall thesis to support integrated limb lengthening over classic lengthening.^28^ In this way, nails and plates seem to simply augment the stability provided by the external fixator that in turn leads to improved outcomes as described.

Certain reservations exist that should be considered with integrated techniques however. The use of additional implants increases the overall cost of performing these techniques when compared with classic techniques. Procedures are more extensive and technically challenging because more devices are involved. The application of these additional devices, such as plates and nails, is contingent on an intact soft-tissue envelope and the absence of deep infection. Such indicative limitations do not apply to classic lengthening with an external fixator alone. The risk of deep intramedullary infection is higher, especially with the use of IM nails, as shown in this study. In particular, we note that LATN and LATP techniques resulted in no deep infection rates. We surmise that implanting internal hardware after removal of the external fixator may be the critical factor in avoiding deep infection in patients undergoing integrated lengthening procedures. Despite this, the overwhelming conclusion is in favor of integrated limb lengthening for all measured outcomes. Even when considering the overall complication burden for both groups, we still observed significant reductions in “problems” (*P* = 0.000) and “sequelae” (*P* = 0.001) in the integrated group.

### Limitations

This study had limitations. We were unable to restrict our inclusion criteria to randomized controlled trials alone because of the lack of suitable publications in the literature in this field. We therefore included retrospective comparative cohort studies that introduces the inherent limitation associated with retrospective analysis. Missing data were a minor issue with a number of studies. Limited data were available regarding objective joint range of motion measurements and subjective patient-reported functional outcome measures meaning that these outcomes could not be included in the final results.

The risk of bias because of heterogeneity was assessed using the *I*^2^ statistic and in each meta-analysis performed, it was found that notable heterogeneity exists that must be considered when interpreting the findings of this study. Funnel plots and Egger tests were used to confirm the absence of any bias because of the small-study effects.

## Conclusions

For all outcome variables, integrated methods of limb lengthening confer a notable advantage over classic limb lengthening methods. Radiographic outcomes, time to union, and time in frame were all improved. The incidence of complications was also reduced notably in the integrated group. This was true on subgroup analysis for all techniques including LATN, LON, and LATP techniques. We suggest the integration of plates and nails with circular frames to improve outcomes in patients requiring limb lengthening procedures.
